# Supplementary material for: Differences in Driving Intention Transitions Caused by Driver’s Emotion Evolutions
Source: Int J Environ Res Public Health. 2020 Sep 23;17(19):6962. doi: 10.3390/ijerph17196962 (PMC7578958; doi:10.3390/ijerph17196962)

The Key Steps of Model Parameter Training

(i). Initialization of the model

Assign the , and with initial values respectively. The assignment of the above parameters was arbitrary under the premise of satisfying the basic probability statistical law.

(ii) Probability calculation based on the forward-backward algorithm

Forward probability calculation of observation sequence:

|  | (S1) |
| --- | --- |

Denote the forward probability as . It is the joint probability of and observation was at the initial moment. For :

|  | (S2) |
| --- | --- |

Calculate the forward probability that the observation sequence was and the hidden state was at the :

|  | (S3) |
| --- | --- |

Backward probability calculation of observation sequence:

|  | (S4) |
| --- | --- |

Denote the backward probability as . It’s the joint probability of and observation was at the initial moment. For :

|  | (S5) |
| --- | --- |

Calculate the backward probability that the observation sequence was after the time and the hidden state was at the time :

|  | (S6) |
| --- | --- |

(ii) Iteration revaluation for parameters based on Baum-Welch algorithm

Determine the log-likelihood function of the probability formula in the first:

|  | (S7) |
| --- | --- |

Where, were the maximized parameters. were the current estimations of Note as:

|  | (S8) |
| --- | --- |

Calculate the three items to the right of the medium number in (S8) by the Lagrange multiplier method. (S9), (S10), and (S11) showed the corresponding Lagrangian function:

|  | (S9) |
| --- | --- |

Where, . was the parameter of the Lagrange multiplier method and the was:

|  | (S10) |
| --- | --- |
|  | (S11) |
|  | (S12) |

Where, and needed to meet the constraint condition and .


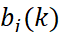

Supplement: Supplementary file 1 [file ijerph-17-06962-s001.zip › Supplementary Materials/Supplementary Material 1.docx]
