# Supplementary material for: Differences in Driving Intention Transitions Caused by Driver’s Emotion Evolutions
Source: Int J Environ Res Public Health. 2020 Sep 23;17(19):6962. doi: 10.3390/ijerph17196962 (PMC7578958; doi:10.3390/ijerph17196962)
Supplement: Supplementary file 1 [file ijerph-17-06962-s001.zip › Supplementary Materials/Supplementary Material 2.docx]

**Table S1.** Probabilities of observation states in different driving intention states.

| **△*d_e_*** | ***d*** | **△*v_e_*** | **Int_1_** | **Int_2_** | **Int_3_** | **△*d_e_*** | ***d*** | **△*v_e_*** | **Int_1_** | **Int_2_** | **Int_3_** | **△*d_e_*** | ***d*** | **△*v_e_*** | **Int_1_** | **Int_2_** | **Int_3_** |
| --- | --- | --- | --- | --- | --- | --- | --- | --- | --- | --- | --- | --- | --- | --- | --- | --- | --- |
| 1 | 1 | 1 | 0.021 | 0.034 | 0.017 | 2 | 1 | 1 | 0.074 | 0.040 | 0.046 | 3 | 1 | 1 | 0.104 | 0.104 | 0.027 |
| 1 | 1 | 2 | 0.028 | 0.019 | 0.014 | 2 | 1 | 2 | 0.029 | 0.045 | 0.042 | 3 | 1 | 2 | 0.084 | 0.082 | 0.031 |
| 1 | 1 | 3 | 0.023 | 0.027 | 0.071 | 2 | 1 | 3 | 0.071 | 0.072 | 0.059 | 3 | 1 | 3 | 0.011 | 0.012 | 0.009 |
| 1 | 2 | 1 | 0.017 | 0.014 | 0.062 | 2 | 2 | 1 | 0.068 | 0.055 | 0.097 | 3 | 2 | 1 | 0.086 | 0.093 | 0.069 |
| 1 | 2 | 2 | 0.035 | 0.023 | 0.030 | 2 | 2 | 2 | 0.005 | 0.006 | 0.011 | 3 | 2 | 2 | 0.014 | 0.007 | 0.011 |
| 1 | 2 | 3 | 0.025 | 0.025 | 0.017 | 2 | 2 | 3 | 0.076 | 0.072 | 0.082 | 3 | 2 | 3 | 0.036 | 0.062 | 0.028 |
| 1 | 3 | 1 | 0.007 | 0.009 | 0.023 | 2 | 3 | 1 | 0.036 | 0.037 | 0.080 | 3 | 3 | 1 | 0.042 | 0.049 | 0.060 |
| 1 | 3 | 2 | 0.015 | 0.009 | 0.022 | 2 | 3 | 2 | 0.005 | 0.009 | 0.022 | 3 | 3 | 2 | 0.018 | 0.006 | 0.011 |
| 1 | 3 | 3 | 0.007 | 0.010 | 0.021 | 2 | 3 | 3 | 0.045 | 0.044 | 0.008 | 3 | 3 | 3 | 0.019 | 0.034 | 0.032 |

**Table S2.** Probabilities of observation states in different HMMs.

| **Emotion** | **△*d_e_*** | ***d*** | **△*v_e_*** | **Int_1_** | **Int_2_** | **Int_3_** | **△*d_e_*** | ***d*** | **△*v_e_*** | **Int_1_** | **Int_2_** | **Int_3_** | **△*d_e_*** | ***d*** | **△*v_e_*** | **Int_1_** | **Int_2_** | **Int_3_** |
| --- | --- | --- | --- | --- | --- | --- | --- | --- | --- | --- | --- | --- | --- | --- | --- | --- | --- | --- |
| Anger | 1 | 1 | 1 | 0.016 | 0.028 | 0.015 | 2 | 1 | 1 | 0.082 | 0.040 | 0.051 | 3 | 1 | 1 | 0.106 | 0.109 | 0.043 |
|  | 1 | 1 | 2 | 0.024 | 0.017 | 0.013 | 2 | 1 | 2 | 0.032 | 0.045 | 0.046 | 3 | 1 | 2 | 0.080 | 0.081 | 0.045 |
|  | 1 | 1 | 3 | 0.020 | 0.025 | 0.068 | 2 | 1 | 3 | 0.079 | 0.073 | 0.066 | 3 | 1 | 3 | 0.011 | 0.012 | 0.013 |
|  | 1 | 2 | 1 | 0.016 | 0.018 | 0.060 | 2 | 2 | 1 | 0.060 | 0.052 | 0.088 | 3 | 2 | 1 | 0.101 | 0.093 | 0.078 |
|  | 1 | 2 | 2 | 0.032 | 0.028 | 0.027 | 2 | 2 | 2 | 0.005 | 0.006 | 0.011 | 3 | 2 | 2 | 0.017 | 0.007 | 0.013 |
|  | 1 | 2 | 3 | 0.024 | 0.032 | 0.016 | 2 | 2 | 3 | 0.073 | 0.074 | 0.080 | 3 | 2 | 3 | 0.041 | 0.061 | 0.031 |
|  | 1 | 3 | 1 | 0.007 | 0.009 | 0.018 | 2 | 3 | 1 | 0.023 | 0.024 | 0.055 | 3 | 3 | 1 | 0.053 | 0.062 | 0.064 |
|  | 1 | 3 | 2 | 0.013 | 0.008 | 0.018 | 2 | 3 | 2 | 0.004 | 0.006 | 0.016 | 3 | 3 | 2 | 0.024 | 0.007 | 0.012 |
|  | 1 | 3 | 3 | 0.007 | 0.010 | 0.017 | 2 | 3 | 3 | 0.031 | 0.031 | 0.006 | 3 | 3 | 3 | 0.021 | 0.039 | 0.031 |
| Surprise | 1 | 1 | 1 | 0.017 | 0.025 | 0.014 | 2 | 1 | 1 | 0.064 | 0.032 | 0.047 | 3 | 1 | 1 | 0.081 | 0.070 | 0.041 |
|  | 1 | 1 | 2 | 0.024 | 0.014 | 0.012 | 2 | 1 | 2 | 0.025 | 0.035 | 0.042 | 3 | 1 | 2 | 0.066 | 0.056 | 0.047 |
|  | 1 | 1 | 3 | 0.021 | 0.022 | 0.065 | 2 | 1 | 3 | 0.060 | 0.056 | 0.059 | 3 | 1 | 3 | 0.009 | 0.008 | 0.013 |
|  | 1 | 2 | 1 | 0.016 | 0.018 | 0.058 | 2 | 2 | 1 | 0.066 | 0.067 | 0.074 | 3 | 2 | 1 | 0.080 | 0.089 | 0.063 |
|  | 1 | 2 | 2 | 0.032 | 0.028 | 0.027 | 2 | 2 | 2 | 0.005 | 0.008 | 0.009 | 3 | 2 | 2 | 0.014 | 0.007 | 0.011 |
|  | 1 | 2 | 3 | 0.022 | 0.030 | 0.014 | 2 | 2 | 3 | 0.080 | 0.094 | 0.067 | 3 | 2 | 3 | 0.034 | 0.060 | 0.026 |
|  | 1 | 3 | 1 | 0.016 | 0.020 | 0.036 | 2 | 3 | 1 | 0.051 | 0.045 | 0.082 | 3 | 3 | 1 | 0.050 | 0.060 | 0.053 |
|  | 1 | 3 | 2 | 0.035 | 0.020 | 0.037 | 2 | 3 | 2 | 0.008 | 0.011 | 0.022 | 3 | 3 | 2 | 0.020 | 0.006 | 0.009 |
|  | 1 | 3 | 3 | 0.017 | 0.023 | 0.035 | 2 | 3 | 3 | 0.065 | 0.055 | 0.008 | 3 | 3 | 3 | 0.021 | 0.040 | 0.028 |
| Fear | 1 | 1 | 1 | 0.019 | 0.027 | 0.018 | 2 | 1 | 1 | 0.056 | 0.030 | 0.043 | 3 | 1 | 1 | 0.085 | 0.075 | 0.039 |
|  | 1 | 1 | 2 | 0.026 | 0.015 | 0.015 | 2 | 1 | 2 | 0.023 | 0.034 | 0.041 | 3 | 1 | 2 | 0.065 | 0.056 | 0.042 |
|  | 1 | 1 | 3 | 0.022 | 0.022 | 0.077 | 2 | 1 | 3 | 0.054 | 0.053 | 0.055 | 3 | 1 | 3 | 0.008 | 0.007 | 0.011 |
|  | 1 | 2 | 1 | 0.017 | 0.019 | 0.065 | 2 | 2 | 1 | 0.073 | 0.070 | 0.073 | 3 | 2 | 1 | 0.081 | 0.086 | 0.054 |
|  | 1 | 2 | 2 | 0.033 | 0.029 | 0.030 | 2 | 2 | 2 | 0.005 | 0.008 | 0.008 | 3 | 2 | 2 | 0.013 | 0.006 | 0.008 |
|  | 1 | 2 | 3 | 0.025 | 0.033 | 0.017 | 2 | 2 | 3 | 0.077 | 0.086 | 0.059 | 3 | 2 | 3 | 0.032 | 0.055 | 0.021 |
|  | 1 | 3 | 1 | 0.018 | 0.022 | 0.047 | 2 | 3 | 1 | 0.053 | 0.047 | 0.086 | 3 | 3 | 1 | 0.048 | 0.059 | 0.043 |
|  | 1 | 3 | 2 | 0.033 | 0.020 | 0.043 | 2 | 3 | 2 | 0.008 | 0.011 | 0.024 | 3 | 3 | 2 | 0.018 | 0.006 | 0.007 |
|  | 1 | 3 | 3 | 0.017 | 0.024 | 0.042 | 2 | 3 | 3 | 0.068 | 0.057 | 0.008 | 3 | 3 | 3 | 0.022 | 0.042 | 0.024 |
| Anxiety | 1 | 1 | 1 | 0.033 | 0.040 | 0.011 | 2 | 1 | 1 | 0.071 | 0.038 | 0.038 | 3 | 1 | 1 | 0.071 | 0.060 | 0.045 |
|  | 1 | 1 | 2 | 0.046 | 0.023 | 0.009 | 2 | 1 | 2 | 0.030 | 0.045 | 0.037 | 3 | 1 | 2 | 0.055 | 0.046 | 0.050 |
|  | 1 | 1 | 3 | 0.039 | 0.033 | 0.045 | 2 | 1 | 3 | 0.073 | 0.073 | 0.052 | 3 | 1 | 3 | 0.008 | 0.007 | 0.014 |
|  | 1 | 2 | 1 | 0.019 | 0.022 | 0.063 | 2 | 2 | 1 | 0.052 | 0.044 | 0.087 | 3 | 2 | 1 | 0.058 | 0.089 | 0.055 |
|  | 1 | 2 | 2 | 0.042 | 0.038 | 0.032 | 2 | 2 | 2 | 0.004 | 0.005 | 0.009 | 3 | 2 | 2 | 0.010 | 0.007 | 0.009 |
|  | 1 | 2 | 3 | 0.030 | 0.042 | 0.018 | 2 | 2 | 3 | 0.054 | 0.054 | 0.068 | 3 | 2 | 3 | 0.025 | 0.061 | 0.023 |
|  | 1 | 3 | 1 | 0.027 | 0.033 | 0.050 | 2 | 3 | 1 | 0.042 | 0.031 | 0.080 | 3 | 3 | 1 | 0.043 | 0.058 | 0.051 |
|  | 1 | 3 | 2 | 0.052 | 0.030 | 0.047 | 2 | 3 | 2 | 0.006 | 0.008 | 0.023 | 3 | 3 | 2 | 0.017 | 0.006 | 0.009 |
|  | 1 | 3 | 3 | 0.025 | 0.033 | 0.042 | 2 | 3 | 3 | 0.049 | 0.036 | 0.007 | 3 | 3 | 3 | 0.018 | 0.038 | 0.026 |
| Helplessness | 1 | 1 | 1 | 0.033 | 0.040 | 0.009 | 2 | 1 | 1 | 0.074 | 0.041 | 0.032 | 3 | 1 | 1 | 0.069 | 0.065 | 0.047 |
|  | 1 | 1 | 2 | 0.053 | 0.026 | 0.008 | 2 | 1 | 2 | 0.029 | 0.046 | 0.029 | 3 | 1 | 2 | 0.055 | 0.052 | 0.054 |
|  | 1 | 1 | 3 | 0.039 | 0.033 | 0.039 | 2 | 1 | 3 | 0.068 | 0.071 | 0.039 | 3 | 1 | 3 | 0.008 | 0.008 | 0.015 |
|  | 1 | 2 | 1 | 0.019 | 0.021 | 0.063 | 2 | 2 | 1 | 0.051 | 0.043 | 0.095 | 3 | 2 | 1 | 0.055 | 0.080 | 0.043 |
|  | 1 | 2 | 2 | 0.043 | 0.037 | 0.034 | 2 | 2 | 2 | 0.004 | 0.005 | 0.011 | 3 | 2 | 2 | 0.010 | 0.006 | 0.008 |
|  | 1 | 2 | 3 | 0.030 | 0.040 | 0.018 | 2 | 2 | 3 | 0.059 | 0.058 | 0.083 | 3 | 2 | 3 | 0.025 | 0.057 | 0.019 |
|  | 1 | 3 | 1 | 0.025 | 0.031 | 0.051 | 2 | 3 | 1 | 0.040 | 0.029 | 0.089 | 3 | 3 | 1 | 0.046 | 0.061 | 0.054 |
|  | 1 | 3 | 2 | 0.049 | 0.029 | 0.049 | 2 | 3 | 2 | 0.006 | 0.007 | 0.025 | 3 | 3 | 2 | 0.017 | 0.006 | 0.009 |
|  | 1 | 3 | 3 | 0.023 | 0.032 | 0.043 | 2 | 3 | 3 | 0.053 | 0.037 | 0.009 | 3 | 3 | 3 | 0.018 | 0.037 | 0.025 |
| Contempt | 1 | 1 | 1 | 0.040 | 0.049 | 0.014 | 2 | 1 | 1 | 0.081 | 0.044 | 0.042 | 3 | 1 | 1 | 0.071 | 0.067 | 0.050 |
|  | 1 | 1 | 2 | 0.047 | 0.023 | 0.010 | 2 | 1 | 2 | 0.032 | 0.049 | 0.038 | 3 | 1 | 2 | 0.059 | 0.056 | 0.059 |
|  | 1 | 1 | 3 | 0.044 | 0.038 | 0.057 | 2 | 1 | 3 | 0.077 | 0.078 | 0.053 | 3 | 1 | 3 | 0.007 | 0.007 | 0.015 |
|  | 1 | 2 | 1 | 0.019 | 0.022 | 0.063 | 2 | 2 | 1 | 0.046 | 0.038 | 0.077 | 3 | 2 | 1 | 0.055 | 0.081 | 0.048 |
|  | 1 | 2 | 2 | 0.037 | 0.034 | 0.029 | 2 | 2 | 2 | 0.003 | 0.004 | 0.009 | 3 | 2 | 2 | 0.009 | 0.006 | 0.008 |
|  | 1 | 2 | 3 | 0.027 | 0.038 | 0.016 | 2 | 2 | 3 | 0.054 | 0.053 | 0.069 | 3 | 2 | 3 | 0.024 | 0.058 | 0.021 |
|  | 1 | 3 | 1 | 0.023 | 0.029 | 0.046 | 2 | 3 | 1 | 0.039 | 0.028 | 0.076 | 3 | 3 | 1 | 0.043 | 0.056 | 0.051 |
|  | 1 | 3 | 2 | 0.047 | 0.027 | 0.045 | 2 | 3 | 2 | 0.006 | 0.007 | 0.021 | 3 | 3 | 2 | 0.018 | 0.006 | 0.010 |
|  | 1 | 3 | 3 | 0.023 | 0.030 | 0.040 | 2 | 3 | 3 | 0.049 | 0.034 | 0.007 | 3 | 3 | 3 | 0.018 | 0.037 | 0.026 |
| Relief | 1 | 1 | 1 | 0.036 | 0.044 | 0.011 | 2 | 1 | 1 | 0.080 | 0.045 | 0.037 | 3 | 1 | 1 | 0.066 | 0.065 | 0.048 |
|  | 1 | 1 | 2 | 0.048 | 0.024 | 0.008 | 2 | 1 | 2 | 0.030 | 0.048 | 0.033 | 3 | 1 | 2 | 0.050 | 0.049 | 0.051 |
|  | 1 | 1 | 3 | 0.046 | 0.039 | 0.050 | 2 | 1 | 3 | 0.074 | 0.078 | 0.046 | 3 | 1 | 3 | 0.008 | 0.008 | 0.016 |
|  | 1 | 2 | 1 | 0.019 | 0.021 | 0.065 | 2 | 2 | 1 | 0.051 | 0.041 | 0.089 | 3 | 2 | 1 | 0.057 | 0.085 | 0.040 |
|  | 1 | 2 | 2 | 0.040 | 0.034 | 0.031 | 2 | 2 | 2 | 0.004 | 0.005 | 0.010 | 3 | 2 | 2 | 0.009 | 0.006 | 0.007 |
|  | 1 | 2 | 3 | 0.029 | 0.037 | 0.017 | 2 | 2 | 3 | 0.059 | 0.057 | 0.078 | 3 | 2 | 3 | 0.023 | 0.054 | 0.015 |
|  | 1 | 3 | 1 | 0.026 | 0.031 | 0.051 | 2 | 3 | 1 | 0.040 | 0.030 | 0.087 | 3 | 3 | 1 | 0.041 | 0.051 | 0.051 |
|  | 1 | 3 | 2 | 0.047 | 0.027 | 0.045 | 2 | 3 | 2 | 0.006 | 0.007 | 0.024 | 3 | 3 | 2 | 0.018 | 0.006 | 0.010 |
|  | 1 | 3 | 3 | 0.026 | 0.033 | 0.045 | 2 | 3 | 3 | 0.050 | 0.036 | 0.008 | 3 | 3 | 3 | 0.019 | 0.038 | 0.029 |
| Pleasure | 1 | 1 | 1 | 0.037 | 0.046 | 0.012 | 2 | 1 | 1 | 0.080 | 0.044 | 0.039 | 3 | 1 | 1 | 0.067 | 0.064 | 0.047 |
|  | 1 | 1 | 2 | 0.051 | 0.026 | 0.010 | 2 | 1 | 2 | 0.030 | 0.047 | 0.034 | 3 | 1 | 2 | 0.057 | 0.053 | 0.056 |
|  | 1 | 1 | 3 | 0.045 | 0.040 | 0.053 | 2 | 1 | 3 | 0.075 | 0.077 | 0.049 | 3 | 1 | 3 | 0.008 | 0.008 | 0.016 |
|  | 1 | 2 | 1 | 0.020 | 0.023 | 0.067 | 2 | 2 | 1 | 0.048 | 0.041 | 0.085 | 3 | 2 | 1 | 0.055 | 0.079 | 0.047 |
|  | 1 | 2 | 2 | 0.037 | 0.033 | 0.029 | 2 | 2 | 2 | 0.003 | 0.005 | 0.009 | 3 | 2 | 2 | 0.009 | 0.006 | 0.008 |
|  | 1 | 2 | 3 | 0.028 | 0.037 | 0.016 | 2 | 2 | 3 | 0.051 | 0.051 | 0.068 | 3 | 2 | 3 | 0.023 | 0.054 | 0.019 |
|  | 1 | 3 | 1 | 0.024 | 0.031 | 0.049 | 2 | 3 | 1 | 0.042 | 0.031 | 0.085 | 3 | 3 | 1 | 0.044 | 0.058 | 0.051 |
|  | 1 | 3 | 2 | 0.049 | 0.029 | 0.047 | 2 | 3 | 2 | 0.006 | 0.008 | 0.023 | 3 | 3 | 2 | 0.019 | 0.007 | 0.009 |
|  | 1 | 3 | 3 | 0.023 | 0.031 | 0.041 | 2 | 3 | 3 | 0.051 | 0.037 | 0.008 | 3 | 3 | 3 | 0.017 | 0.035 | 0.024 |
