# Supplementary material for: Differences in Driving Intention Transitions Caused by Driver’s Emotion Evolutions
Source: Int J Environ Res Public Health. 2020 Sep 23;17(19):6962. doi: 10.3390/ijerph17196962 (PMC7578958; doi:10.3390/ijerph17196962)
Supplement: Supplementary file 1 [file ijerph-17-06962-s001.zip › Supplementary Materials/Supplementary Material 3.docx]

**Table S3.** F-test for probability of driving intentions under different time window

| **F-test for probability of Int1** | | | | | | |
| --- | --- | --- | --- | --- | --- | --- |
| Time window | | 10 seconds | | 5 seconds | 15 seconds | Total |
| $df$ | | 62 | | 62 | 62 | 186 |
| $\overline{x_{i}}$ | | 0.29 | | 0.28 | 0.30 | 0.87 |
| $\sum x_{i}$ | | 17.98 | | 17.38 | 18.34 | 53.71 |
| $\frac{(\sum x_{i})^{2}}{df}$ | | 5.21 | | 4.87 | 5.43 | 15.52 |
| $\sum x_{i}^{2}$ | | 5.36 | | 5.07 | 5.75 | 16.18 |
| Sources of variation | Quadratic sum | $df$ | Mean square error | F value | F_α_(7, 488) | Sig. |
| Factor | 0.01 | 2 | 0.0038 | 1.047 | F_0.1_=1.729 | - ^1^ |
| error | 0.66 | 183 | 0.0036 |  | F_0.05_=2.208 |  |
| Total | 0.67 | 185 |  |  | F_0.01_=2.676 |  |
| **F-test for probability of Int 2** | | | | | | |
| Time window | | 10 seconds | | 5 seconds | 15 seconds | Total |
| $df$ | | 62 | | 62 | 62 | 186 |
| $\overline{x_{i}}$ | | 0.47 | | 0.47 | 0.47 | 1.41 |
| $\sum x_{i}$ | | 29.13 | | 29.22 | 29.36 | 87.70 |
| $\frac{(\sum x_{i})^{2}}{df}$ | | 13.68 | | 13.77 | 13.90 | 41.35 |
| $\sum x_{i}^{2}$ | | 13.84 | | 14.01 | 14.36 | 42.21 |
| Sources of variation | Quadratic sum | $df$ | Mean square error | F value | F_α_(7, 488) | Sig. |
| Factor | 0.00 | 2 | 0.0002 | 0.045 | F0.1=1.729 | - |
| error | 0.86 | 183 | 0.0047 |  | F0.05=2.208 |  |
| Total | 0.86 | 185 |  |  | F0.01=2.676 |  |
| **F-test for probability of Int 3** | | | | | | |
| Time window | | 10 seconds | | 5 seconds | 15 seconds | Total |
| $df$ | | 62 | | 62 | 62 | 186 |
| $\overline{x_{i}}$ | | 0.25 | | 0.25 | 0.23 | 0.73 |
| $\sum x_{i}$ | | 15.50 | | 15.40 | 14.30 | 45.20 |
| $\frac{(\sum x_{i})^{2}}{df}$ | | 3.88 | | 3.82 | 3.30 | 11.00 |
| $\sum x_{i}^{2}$ | | 4.23 | | 4.28 | 4.04 | 12.54 |
| Sources of variation | Quadratic sum | 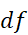 | Mean square error | F value | F_α_(7, 488) | Sig. |
| Factor | 0.01 | 2 | 0.0071 | 0.845 | F0.1=1.729 | - |
| error | 1.55 | 183 | 0.0085 |  | F0.05=2.208 |  |
| Total | 1.56 | 185 |  |  | F0.01=2.676 |  |

^1^ - represented non-significant.

The results of the F-test showed that there is no significant difference in the probability distribution of Int1, Int2, and Int3 under different time Windows.

**Table S4.** F-test for transition probability of driving intentions under different time window

| **F-test for transition probability of Int1🡪 Int1** | | | | | | |
| --- | --- | --- | --- | --- | --- | --- |
| Time window | | 10 seconds | | 5 seconds | 15 seconds | Total |
| $df$ | | 62 | | 62 | 62 | 186 |
| $\overline{x_{i}}$ | | 0.26 | | 0.25 | 0.25 | 0.76 |
| $\sum x_{i}$ | | 16.07 | | 15.80 | 15.48 | 47.35 |
| $\frac{(\sum x_{i})^{2}}{df}$ | | 4.17 | | 4.03 | 3.87 | 12.06 |
| $\sum x_{i}^{2}$ | | 4.28 | | 4.15 | 4.00 | 12.43 |
| Sources of variation | Quadratic sum | $df$ | Mean square error | F value | F_α_(7, 488) | Sig. |
| Factor | 0.003 | 2 | 0.0014 | 0.692 | F_0.1_=1.729 | - ^1^ |
| error | 0.370 | 183 | 0.0020 |  | F_0.05_=2.208 |  |
| Total | 0.373 | 185 |  |  | F_0.01_=2.676 |  |
| **F-test for transition probability of Int1🡪 Int2** | | | | | | |
| Time window | | 10 seconds | | 5 seconds | 15 seconds | Total |
| $df$ | | 62 | | 62 | 62 | 186 |
| $\overline{x_{i}}$ | | 0.52 | | 0.52 | 0.51 | 1.55 |
| $\sum x_{i}$ | | 32.34 | | 32.02 | 31.75 | 96.10 |
| $\frac{(\sum x_{i})^{2}}{df}$ | | 16.86 | | 16.54 | 16.25 | 49.66 |
| $\sum x_{i}^{2}$ | | 17.41 | | 17.11 | 16.80 | 51.32 |
| Sources of variation | Quadratic sum | $df$ | Mean square error | F value | F_α_(7, 488) | Sig. |
| Factor | 0.003 | 2 | 0.0014 | 0.154 | F0.1=1.729 | - |
| error | 1.667 | 183 | 0.0091 |  | F0.05=2.208 |  |
| Total | 1.670 | 185 |  |  | F0.01=2.676 |  |
| **F-test for transition probability of Int1🡪 Int3** | | | | | | |
| Time window | | 10 seconds | | 5 seconds | 15 seconds | Total |
| $df$ | | 62 | | 62 | 62 | 186 |
| $\overline{x_{i}}$ | | 0.22 | | 0.23 | 0.24 | 0.69 |
| $\sum x_{i}$ | | 13.59 | | 14.18 | 14.77 | 42.55 |
| $\frac{(\sum x_{i})^{2}}{df}$ | | 2.98 | | 3.24 | 3.52 | 9.74 |
| $\sum x_{i}^{2}$ | | 3.69 | | 3.83 | 4.23 | 11.75 |
| Sources of variation | Quadratic sum | $df$ | Mean square error | F value | F_α_(7, 488) | Sig. |
| Factor | 0.011 | 2 | 0.0056 | 0.511 | F0.1=1.729 | - |
| error | 2.007 | 183 | 0.0110 |  | F0.05=2.208 |  |
| Total | 2.018 | 185 |  |  | F0.01=2.676 |  |
| **F-test for transition probability of Int2🡪 Int1** | | | | | | |
| Time window | | 10 seconds | | 5 seconds | 15 seconds | Total |
| $df$ | | 62 | | 62 | 62 | 186 |
| $\overline{x_{i}}$ | | 0.34 | | 0.34 | 0.34 | 1.02 |
| $\sum x_{i}$ | | 21.37 | | 20.99 | 20.93 | 63.29 |
| $\frac{(\sum x_{i})^{2}}{df}$ | | 7.36 | | 7.11 | 7.07 | 21.54 |
| $\sum x_{i}^{2}$ | | 7.58 | | 7.34 | 7.32 | 22.24 |
| Sources of variation | Quadratic sum | $df$ | Mean square error | F value | F_α_(7, 488) | Sig. |
| Factor | 0.002 | 2 | 0.0009 | 0.236 | F_0.1_=1.729 | - ^1^ |
| error | 0.700 | 183 | 0.0038 |  | F_0.05_=2.208 |  |
| Total | 0.702 | 185 |  |  | F_0.01_=2.676 |  |
| **F-test for transition probability of Int2🡪 Int2** | | | | | | |
| Time window | | 10 seconds | | 5 seconds | 15 seconds | Total |
| $df$ | | 62 | | 62 | 62 | 186 |
| $\overline{x_{i}}$ | | 0.37 | | 0.37 | 0.36 | 1.11 |
| $\sum x_{i}$ | | 23.18 | | 22.95 | 22.48 | 68.61 |
| $\frac{(\sum x_{i})^{2}}{df}$ | | 8.67 | | 8.49 | 8.15 | 25.31 |
| $\sum x_{i}^{2}$ | | 8.89 | | 8.72 | 8.39 | 26.00 |
| Sources of variation | Quadratic sum | $df$ | Mean square error | F value | F_α_(7, 488) | Sig. |
| Factor | 0.004 | 2 | 0.0021 | 0.546 | F0.1=1.729 | - |
| error | 0.688 | 183 | 0.0038 |  | F0.05=2.208 |  |
| Total | 0.692 | 185 |  |  | F0.01=2.676 |  |
| **F-test for transition probability of Int2🡪 Int3** | | | | | | |
| Time window | | 10 seconds | | 5 seconds | 15 seconds | Total |
| $df$ | | 62 | | 62 | 62 | 186 |
| $\overline{x_{i}}$ | | 0.28 | | 0.29 | 0.30 | 0.87 |
| $\sum x_{i}$ | | 17.45 | | 18.06 | 18.59 | 54.10 |
| $\frac{(\sum x_{i})^{2}}{df}$ | | 4.91 | | 5.26 | 5.57 | 15.74 |
| $\sum x_{i}^{2}$ | | 5.26 | | 5.77 | 5.91 | 16.94 |
| Sources of variation | Quadratic sum | $df$ | Mean square error | F value | F_α_(7, 488) | Sig. |
| Factor | 0.010 | 2 | 0.0052 | 0.799 | F0.1=1.729 | - |
| error | 1.198 | 183 | 0.0065 |  | F0.05=2.208 |  |
| Total | 1.208 | 185 |  |  | F0.01=2.676 |  |
| **F-test for transition probability of Int3🡪 Int1** | | | | | | |
| Time window | | 10 seconds | | 5 seconds | 15 seconds | Total |
| $df$ | | 62 | | 62 | 62 | 186 |
| $\overline{x_{i}}$ | | 0.22 | | 0.22 | 0.22 | 0.66 |
| $\sum x_{i}$ | | 13.85 | | 13.58 | 13.56 | 40.99 |
| $\frac{(\sum x_{i})^{2}}{df}$ | | 3.09 | | 2.98 | 2.97 | 9.03 |
| $\sum x_{i}^{2}$ | | 3.16 | | 3.05 | 3.04 | 9.25 |
| Sources of variation | Quadratic sum | $df$ | Mean square error | F value | F_α_(7, 488) | Sig. |
| Factor | 0.001 | 2 | 0.0004 | 0.336 | F_0.1_=1.729 | - ^1^ |
| error | 0.220 | 183 | 0.0012 |  | F_0.05_=2.208 |  |
| Total | 0.221 | 185 |  |  | F_0.01_=2.676 |  |
| **F-test for transition probability of Int3🡪 Int2** | | | | | | |
| Time window | | 10 seconds | | 5 seconds | 15 seconds | Total |
| $df$ | | 62 | | 62 | 62 | 186 |
| $\overline{x_{i}}$ | | 0.56 | | 0.56 | 0.55 | 1.67 |
| $\sum x_{i}$ | | 34.94 | | 34.49 | 34.23 | 103.66 |
| $\frac{(\sum x_{i})^{2}}{df}$ | | 19.69 | | 19.19 | 18.90 | 57.78 |
| $\sum x_{i}^{2}$ | | 20.22 | | 19.72 | 19.44 | 59.38 |
| Sources of variation | Quadratic sum | $df$ | Mean square error | F value | F_α_(7, 488) | Sig. |
| Factor | 0.004 | 2 | 0.0020 | 0.233 | F0.1=1.729 | - |
| error | 1.605 | 183 | 0.0088 |  | F0.05=2.208 |  |
| Total | 1.609 | 185 |  |  | F0.01=2.676 |  |
| **F-test for transition probability of Int3🡪 Int3** | | | | | | |
| Time window | | 10 seconds | | 5 seconds | 15 seconds | Total |
| $df$ | | 62 | | 62 | 62 | 186 |
| $\overline{x_{i}}$ | | 0.21 | | 0.22 | 0.23 | 0.67 |
| $\sum x_{i}$ | | 13.22 | | 13.92 | 14.21 | 41.35 |
| $\frac{(\sum x_{i})^{2}}{df}$ | | 2.82 | | 3.13 | 3.26 | 9.20 |
| $\sum x_{i}^{2}$ | | 3.48 | | 3.70 | 3.91 | 11.08 |
| Sources of variation | Quadratic sum | $df$ | Mean square error | F value | F_α_(7, 488) | Sig. |
| Factor | 0.008 | 2 | 0.0042 | 0.406 | F0.1=1.729 | - |
| error | 1.882 | 183 | 0.0103 |  | F0.05=2.208 |  |
| Total | 1.891 | 185 |  |  | F0.01=2.676 |  |

^1^ - represented non-significant.

The results of the F-test showed that there is no significant difference in the transition probability of Int1🡪Int1, Int1🡪Int1, Int1🡪Int2, Int1🡪Int3, Int2🡪Int1, Int2🡪Int2, Int2🡪Int3, Int3🡪Int1, Int3🡪Int2, and Int3🡪Int3 under different time Windows.

**Table S5.** F-test for probability of observation states under different time window

| **△*d_e_*** | ***d*** | **△*v_e_*** | **Int_1_** | | | | | **Int_2_** | | | | | **Int_3_** | | | | |
| --- | --- | --- | --- | --- | --- | --- | --- | --- | --- | --- | --- | --- | --- | --- | --- | --- | --- |
|  |  |  | 10 s | 5 s | 15 s | F | Sig | 10 s | 5 s | 15 s | F | Sig | 10 s | 5 s | 15 s | F | Sig |
| 1 | 1 | 1 | 0.021 | 0.020 | 0.020 | 1.099 | - | 0.034 | 0.034 | 0.032 | 1.178 | - | 0.017 | 0.016 | 0.016 | 0.888 | - |
| 1 | 1 | 2 | 0.028 | 0.030 | 0.026 | 1.141 | - | 0.019 | 0.019 | 0.019 | 0.703 | - | 0.014 | 0.015 | 0.014 | 1.164 | - |
| 1 | 1 | 3 | 0.023 | 0.025 | 0.022 | 1.46 | - | 0.027 | 0.028 | 0.026 | 0.91 | - | 0.071 | 0.072 | 0.078 | 1.417 | - |
| 1 | 2 | 1 | 0.017 | 0.016 | 0.015 | 1.203 | - | 0.014 | 0.013 | 0.014 | 0.776 | - | 0.062 | 0.063 | 0.068 | 0.467 | - |
| 1 | 2 | 2 | 0.035 | 0.032 | 0.033 | 0.445 | - | 0.023 | 0.022 | 0.022 | 0.432 | - | 0.030 | 0.030 | 0.027 | 0.943 | - |
| 1 | 2 | 3 | 0.025 | 0.025 | 0.028 | 0.484 | - | 0.025 | 0.027 | 0.024 | 1.361 | - | 0.017 | 0.016 | 0.018 | 0.907 | - |
| 1 | 3 | 1 | 0.007 | 0.007 | 0.007 | 0.429 | - | 0.009 | 0.009 | 0.009 | 1.296 | - | 0.023 | 0.024 | 0.020 | 1.498 | - |
| 1 | 3 | 2 | 0.015 | 0.016 | 0.014 | 0.566 | - | 0.009 | 0.008 | 0.009 | 0.798 | - | 0.022 | 0.022 | 0.022 | 1.494 | - |
| 1 | 3 | 3 | 0.007 | 0.007 | 0.006 | 0.688 | - | 0.010 | 0.011 | 0.009 | 1.397 | - | 0.021 | 0.022 | 0.019 | 1.495 | - |
| 2 | 1 | 1 | 0.074 | 0.080 | 0.081 | 0.913 | - | 0.040 | 0.043 | 0.040 | 0.5 | - | 0.046 | 0.049 | 0.049 | 0.996 | - |
| 2 | 1 | 2 | 0.029 | 0.029 | 0.028 | 0.583 | - | 0.045 | 0.048 | 0.048 | 1.099 | - | 0.042 | 0.044 | 0.038 | 1.309 | - |
| 2 | 1 | 3 | 0.071 | 0.065 | 0.064 | 0.58 | - | 0.072 | 0.067 | 0.075 | 0.797 | - | 0.059 | 0.054 | 0.064 | 1.379 | - |
| 2 | 2 | 1 | 0.068 | 0.071 | 0.066 | 1.192 | - | 0.055 | 0.058 | 0.061 | 1.497 | - | 0.097 | 0.091 | 0.095 | 1.405 | - |
| 2 | 2 | 2 | 0.005 | 0.005 | 0.005 | 0.999 | - | 0.006 | 0.007 | 0.006 | 0.943 | - | 0.011 | 0.012 | 0.011 | 1.175 | - |
| 2 | 2 | 3 | 0.076 | 0.075 | 0.081 | 1.408 | - | 0.072 | 0.073 | 0.064 | 1.212 | - | 0.082 | 0.075 | 0.076 | 1.342 | - |
| 2 | 3 | 1 | 0.036 | 0.039 | 0.034 | 1.295 | - | 0.037 | 0.035 | 0.037 | 0.625 | - | 0.080 | 0.076 | 0.072 | 0.695 | - |
| 2 | 3 | 2 | 0.005 | 0.005 | 0.005 | 1.494 | - | 0.009 | 0.010 | 0.010 | 0.995 | - | 0.022 | 0.024 | 0.023 | 1.577 | - |
| 2 | 3 | 3 | 0.045 | 0.044 | 0.047 | 0.871 | - | 0.044 | 0.041 | 0.046 | 0.671 | - | 0.008 | 0.008 | 0.007 | 1.35 | - |
| 3 | 1 | 1 | 0.104 | 0.101 | 0.115 | 1.585 | - | 0.104 | 0.095 | 0.112 | 0.664 | - | 0.027 | 0.028 | 0.026 | 0.981 | - |
| 3 | 1 | 2 | 0.084 | 0.081 | 0.084 | 1.167 | - | 0.082 | 0.080 | 0.086 | 1.516 | - | 0.031 | 0.031 | 0.028 | 1.027 | - |
| 3 | 1 | 3 | 0.011 | 0.010 | 0.010 | 1.194 | - | 0.012 | 0.012 | 0.012 | 0.61 | - | 0.009 | 0.009 | 0.009 | 0.884 | - |
| 3 | 2 | 1 | 0.086 | 0.090 | 0.086 | 1.03 | - | 0.093 | 0.087 | 0.100 | 0.799 | - | 0.069 | 0.066 | 0.068 | 1.303 | - |
| 3 | 2 | 2 | 0.014 | 0.013 | 0.013 | 0.634 | - | 0.007 | 0.007 | 0.006 | 1.444 | - | 0.011 | 0.012 | 0.012 | 1.374 | - |
| 3 | 2 | 3 | 0.036 | 0.038 | 0.039 | 1.131 | - | 0.062 | 0.064 | 0.063 | 1.2 | - | 0.028 | 0.028 | 0.027 | 1.445 | - |
| 3 | 3 | 1 | 0.042 | 0.045 | 0.044 | 1.336 | - | 0.049 | 0.052 | 0.051 | 0.584 | - | 0.060 | 0.065 | 0.067 | 0.936 | - |
| 3 | 3 | 2 | 0.018 | 0.019 | 0.018 | 0.529 | - | 0.006 | 0.006 | 0.006 | 0.828 | - | 0.011 | 0.011 | 0.012 | 0.718 | - |
| 3 | 3 | 3 | 0.019 | 0.017 | 0.021 | 1.074 | - | 0.034 | 0.035 | 0.034 | 1.174 | - | 0.032 | 0.033 | 0.034 | 1.293 | - |

^1^ - represented non-significant.

The results of the F-test showed that there is no significant difference in the probability of observation states under different time Windows.
