# Supplementary material for: Differences in Driving Intention Transitions Caused by Driver’s Emotion Evolutions
Source: Int J Environ Res Public Health. 2020 Sep 23;17(19):6962. doi: 10.3390/ijerph17196962 (PMC7578958; doi:10.3390/ijerph17196962)
Supplement: Supplementary file 1 [file ijerph-17-06962-s001.zip › Supplementary Materials/Supplementary Material 4.docx]

**Table S6.** F-test results for the transition probability of driving intention under different emotions.

| **F-test for transition probability of Int1🡪 Int1** | | | | | | | | | |
| --- | --- | --- | --- | --- | --- | --- | --- | --- | --- |
| Emotion | Em1 | Em2 | Em3 | Em4 | Em5 | Em6 | Em7 | Em8 | Total |
| $df$ | 62 | 62 | 62 | 62 | 62 | 62 | 62 | 62 | 496 |
| $\overline{x_{i}}$ | 0.16 | 0.27 | 0.32 | 0.33 | 0.31 | 0.20 | 0.26 | 0.23 | 2.09 |
| $\sum x_{i}$ | 9.81 | 16.71 | 17.93 | 18.45 | 17.36 | 11.81 | 15.87 | 14.29 | 122.22 |
| $\frac{(\sum x_{i})^{2}}{df}$ | 1.55 | 4.50 | 5.19 | 5.49 | 4.86 | 2.25 | 4.06 | 3.29 | 31.19 |
| $\sum x_{i}^{2}$ | 1.62 | 4.71 | 5.30 | 5.58 | 4.95 | 2.39 | 4.19 | 3.38 | 32.12 |
| Sources of variation | Quadratic sum | | $df$ | Mean square error | | F value | F_α_(7, 488) | | Sig. |
| Factor | 1.08 | | 7 | 0.15 | | 81.37 | F_0.1_=1.729 | | ***^1^ |
| error | 0.92 | | 488 | 0.00 | |  | F_0.05_=2.208 | |  |
| Total | 2.00 | | 495 |  | |  | F_0.01_=2.676 | |  |
| **F-test for transition probability of Int1🡪 Int2** | | | | | | | | | |
| Emotion | Em1 | Em2 | Em3 | Em4 | Em5 | Em6 | Em7 | Em8 | Total |
| $df$ | 62 | 62 | 62 | 62 | 62 | 62 | 62 | 62 | 496 |
| $\overline{x_{i}}$ | 0.48 | 0.40 | 0.53 | 0.39 | 0.50 | 0.51 | 0.51 | 0.52 | 3.84 |
| $\sum x_{i}$ | 28.67 | 24.50 | 29.69 | 22.27 | 27.84 | 29.73 | 30.42 | 31.84 | 224.97 |
| $\frac{(\sum x_{i})^{2}}{df}$ | 13.26 | 9.68 | 14.22 | 8.00 | 12.50 | 14.26 | 14.93 | 16.35 | 103.20 |
| $\sum x_{i}^{2}$ | 13.80 | 10.09 | 14.57 | 8.18 | 12.77 | 14.56 | 15.30 | 16.80 | 106.06 |
| Sources of variation | Quadratic sum | | $df$ | Mean square error | | F value | F_α_(7, 488) | | Sig. |
| Factor | 1.16 | | 7 | 0.17 | | 28.30 | F_0.1_=1.729 | | ***^1^ |
| error | 2.86 | | 488 | 0.01 | |  | F_0.05_=2.208 | |  |
| Total | 4.02 | | 495 |  | |  | F_0.01_=2.676 | |  |
| **F-test for transition probability of Int1🡪 Int3** | | | | | | | | | |
| Emotion | Em1 | Em2 | Em3 | Em4 | Em5 | Em6 | Em7 | Em8 | Total |
| $df$ | 62 | 62 | 62 | 62 | 62 | 62 | 62 | 62 | 496 |
| $\overline{x_{i}}$ | 0.36 | 0.33 | 0.15 | 0.28 | 0.19 | 0.29 | 0.23 | 0.25 | 2.08 |
| $\sum x_{i}$ | 23.52 | 20.79 | 14.38 | 21.28 | 16.80 | 20.46 | 15.71 | 15.87 | 148.80 |
| $\frac{(\sum x_{i})^{2}}{df}$ | 8.92 | 6.97 | 3.34 | 7.30 | 4.55 | 6.75 | 3.98 | 4.06 | 45.88 |
| $\sum x_{i}^{2}$ | 9.52 | 7.61 | 3.73 | 7.53 | 4.98 | 7.15 | 4.44 | 4.62 | 49.60 |
| Sources of variation | Quadratic sum | | $df$ | Mean square error | | F value | F_α_(7, 488) | | Sig. |
| Factor | 1.23 | | 7 | 0.18 | | 23.11 | F_0.1_=1.729 | | ***^1^ |
| error | 3.72 | | 488 | 0.01 | |  | F_0.05_=2.208 | |  |
| Total | 4.95 | | 495 |  | |  | F_0.01_=2.676 | |  |
| **F-test for transition probability of Int2🡪 Int1** | | | | | | | | | |
| Emotion | Em1 | Em2 | Em3 | Em4 | Em5 | Em6 | Em7 | Em8 | Total |
| $df$ | 62 | 62 | 62 | 62 | 62 | 62 | 62 | 62 | 496 |
| $\overline{x_{i}}$ | 0.21 | 0.28 | 0.35 | 0.30 | 0.33 | 0.23 | 0.32 | 0.29 | 2.31 |
| $\sum x_{i}$ | 12.96 | 17.37 | 19.99 | 17.42 | 18.94 | 13.87 | 20.01 | 17.93 | 138.49 |
| $\frac{(\sum x_{i})^{2}}{df}$ | 2.71 | 4.87 | 6.45 | 4.90 | 5.79 | 3.10 | 6.46 | 5.19 | 39.45 |
| $\sum x_{i}^{2}$ | 2.82 | 5.09 | 6.62 | 5.01 | 5.92 | 3.27 | 6.66 | 5.35 | 40.75 |
| Sources of variation | Quadratic sum | | $df$ | Mean square error | | F value | F_α_(7, 488) | | Sig. |
| Factor | 0.78 | | 7 | 0.11 | | 41.65 | F_0.1_=1.729 | | ***^1^ |
| error | 1.30 | | 488 | 0.00 | |  | F_0.05_=2.208 | |  |
| Total | 2.08 | | 495 |  | |  | F_0.01_=2.676 | |  |
| **F-test for transition probability of Int2🡪 Int2** | | | | | | | | | |
| Emotion | Em1 | Em2 | Em3 | Em4 | Em5 | Em6 | Em7 | Em8 | Total |
| $df$ | 62 | 62 | 62 | 62 | 62 | 62 | 62 | 62 | 496 |
| $\overline{x_{i}}$ | 0.44 | 0.47 | 0.44 | 0.40 | 0.49 | 0.46 | 0.41 | 0.43 | 3.54 |
| $\sum x_{i}$ | 27.36 | 30.04 | 26.01 | 22.84 | 29.14 | 26.21 | 24.96 | 25.27 | 211.84 |
| $\frac{(\sum x_{i})^{2}}{df}$ | 12.08 | 14.56 | 10.91 | 8.42 | 13.70 | 11.08 | 10.05 | 10.30 | 91.08 |
| $\sum x_{i}^{2}$ | 12.48 | 15.10 | 11.14 | 8.62 | 14.13 | 11.37 | 10.30 | 10.54 | 93.69 |
| Sources of variation | Quadratic sum | | $df$ | Mean square error | | F value | F_α_(7, 488) | | Sig. |
| Factor | 0.61 | | 7 | 0.09 | | 16.38 | F_0.1_=1.729 | | ***^1^ |
| error | 2.60 | | 488 | 0.01 | |  | F_0.05_=2.208 | |  |
| Total | 3.21 | | 495 |  | |  | F_0.01_=2.676 | |  |
| **F-test for transition probability of Int2🡪 Int3** | | | | | | | | | |
| Emotion | Em1 | Em2 | Em3 | Em4 | Em5 | Em6 | Em7 | Em8 | Total |
| $df$ | 0.35 | 0.25 | 0.21 | 0.30 | 0.18 | 0.31 | 0.27 | 0.28 | 2.15 |
| $\overline{x_{i}}$ | 21.68 | 14.58 | 16.00 | 21.74 | 13.91 | 21.92 | 17.04 | 18.80 | 145.67 |
| $\sum x_{i}$ | 7.58 | 3.43 | 4.13 | 7.62 | 3.12 | 7.75 | 4.68 | 5.70 | 44.02 |
| $\frac{(\sum x_{i})^{2}}{df}$ | 8.09 | 4.19 | 4.53 | 7.88 | 3.72 | 8.08 | 5.20 | 6.16 | 47.86 |
| $\sum x_{i}^{2}$ | 0.35 | 0.25 | 0.21 | 0.30 | 0.18 | 0.31 | 0.27 | 0.28 | 2.15 |
| Sources of variation | Quadratic sum | | $df$ | Mean square error | | F value | F_α_(7, 488) | | Sig. |
| Factor | 1.23 | | 7 | 0.18 | | 22.36 | F_0.1_=1.729 | | ***^1^ |
| error | 3.84 | | 488 | 0.01 | |  | F_0.05_=2.208 | |  |
| Total | 5.08 | | 495 |  | |  | F_0.01_=2.676 | |  |
| **F-test for transition probability of Int3🡪 Int1** | | | | | | | | | |
| Emotion | Em1 | Em2 | Em3 | Em4 | Em5 | Em6 | Em7 | Em8 | Total |
| $df$ | 0.16 | 0.23 | 0.29 | 0.26 | 0.25 | 0.20 | 0.22 | 0.18 | 1.79 |
| $\overline{x_{i}}$ | 9.81 | 13.82 | 16.41 | 14.58 | 14.39 | 13.30 | 13.73 | 11.58 | 107.62 |
| $\sum x_{i}$ | 1.55 | 3.08 | 4.34 | 3.43 | 3.34 | 2.85 | 3.04 | 2.16 | 23.80 |
| $\frac{(\sum x_{i})^{2}}{df}$ | 1.62 | 3.21 | 4.46 | 3.53 | 3.43 | 2.96 | 3.11 | 2.22 | 24.55 |
| $\sum x_{i}^{2}$ | 0.16 | 0.23 | 0.29 | 0.26 | 0.25 | 0.20 | 0.22 | 0.18 | 1.79 |
| Sources of variation | Quadratic sum | | $df$ | Mean square error | | F value | F_α_(7, 488) | | Sig. |
| Factor | 0.45 | | 7 | 0.06 | | 41.92 | F_0.1_=1.729 | | ***^1^ |
| error | 0.75 | | 488 | 0.00 | |  | F_0.05_=2.208 | |  |
| Total | 1.20 | | 495 |  | |  | F_0.01_=2.676 | |  |
| **F-test for transition probability of Int3🡪 Int2** | | | | | | | | | |
| Emotion | Em1 | Em2 | Em3 | Em4 | Em5 | Em6 | Em7 | Em8 | Total |
| $df$ | 0.33 | 0.41 | 0.50 | 0.41 | 0.52 | 0.51 | 0.58 | 0.56 | 3.82 |
| $\overline{x_{i}}$ | 21.04 | 26.47 | 29.11 | 22.67 | 29.80 | 29.63 | 36.34 | 35.01 | 230.07 |
| $\sum x_{i}$ | 7.14 | 11.30 | 13.67 | 8.29 | 14.32 | 14.16 | 21.30 | 19.77 | 109.95 |
| $\frac{(\sum x_{i})^{2}}{df}$ | 7.44 | 11.68 | 14.07 | 8.54 | 14.79 | 14.65 | 21.72 | 20.32 | 113.20 |
| $\sum x_{i}^{2}$ | 0.33 | 0.41 | 0.50 | 0.41 | 0.52 | 0.51 | 0.58 | 0.56 | 3.82 |
| Sources of variation | Quadratic sum | | $df$ | Mean square error | | F value | F_α_(7, 488) | | Sig. |
| Factor | 3.23 | | 7 | 0.46 | | 69.29 | F_0.1_=1.729 | | ***^1^ |
| error | 3.25 | | 488 | 0.01 | |  | F_0.05_=2.208 | |  |
| Total | 6.48 | | 495 |  | |  | F_0.01_=2.676 | |  |
| **F-test for transition probability of Int3🡪 Int3** | | | | | | | | | |
| Emotion | Em1 | Em2 | Em3 | Em4 | Em5 | Em6 | Em7 | Em8 | Total |
| $df$ | 0.51 | 0.36 | 0.21 | 0.33 | 0.23 | 0.29 | 0.20 | 0.26 | 2.39 |
| $\overline{x_{i}}$ | 31.14 | 21.71 | 16.48 | 24.74 | 17.81 | 19.07 | 11.93 | 15.41 | 158.31 |
| $\sum x_{i}$ | 15.65 | 7.60 | 4.38 | 9.87 | 5.12 | 5.87 | 2.30 | 3.83 | 54.61 |
| $\frac{(\sum x_{i})^{2}}{df}$ | 15.98 | 8.14 | 4.87 | 10.19 | 5.71 | 6.44 | 2.73 | 4.43 | 58.49 |
| $\sum x_{i}^{2}$ | 0.51 | 0.36 | 0.21 | 0.33 | 0.23 | 0.29 | 0.20 | 0.26 | 2.39 |
| Sources of variation | Quadratic sum | | $df$ | Mean square error | | F value | F_α_(7, 488) | | Sig. |
| Factor | 4.09 | | 7 | 0.58 | | 73.61 | F_0.1_=1.729 | | ***^1^ |
| error | 3.87 | | 488 | 0.01 | |  | F_0.05_=2.208 | |  |
| Total | 7.96 | | 495 |  | |  | F_0.01_=2.676 | |  |

^1^ *** represented the significance level was 0.01, ** represented the significance level was 0.05, * represented the significance level was 0.1, - represented non-significant.

The results of the F-test showed that the eight emotions had significantly different effects on the transition probabilities of Int1🡪Int1, Int1🡪Int2, Int1🡪Int3, Int2🡪Int1, Int2🡪Int2, Int2🡪Int3, Int3🡪Int1, Int3🡪Int2, and Int3🡪Int3.
