# Supplementary material for: Differences in Driving Intention Transitions Caused by Driver’s Emotion Evolutions
Source: Int J Environ Res Public Health. 2020 Sep 23;17(19):6962. doi: 10.3390/ijerph17196962 (PMC7578958; doi:10.3390/ijerph17196962)
Supplement: Supplementary file 1 [file ijerph-17-06962-s001.zip › Supplementary Materials/Supplementary Material 5.docx]

**Table S7.** Probability order of eight emotions in different states of intention transfer

| Int1🡪 Int1 | | | | | | | | |
| --- | --- | --- | --- | --- | --- | --- | --- | --- |
| Emotion | Em1 | Em2 | Em3 | Em4 | Em5 | Em6 | Em7 | Em8 |
| *r_i_* ^1^ | 8 | 4 | 2 | 1 | 3 | 7 | 5 | 6 |
| Int1🡪 Int2 | | | | | | | | |
| Emotion | Em1 | Em2 | Em3 | Em4 | Em5 | Em6 | Em7 | Em8 |
| *r_i_* | 6 | 7 | 1 | 8 | 5 | 3 | 4 | 2 |
| Int1🡪 Int3 | | | | | | | | |
| Emotion | Em1 | Em2 | Em3 | Em4 | Em5 | Em6 | Em7 | Em8 |
| *r_i_* | 1 | 2 | 8 | 4 | 7 | 3 | 6 | 5 |
| Int2🡪 Int1 | | | | | | | | |
| Emotion | Em1 | Em2 | Em3 | Em4 | Em5 | Em6 | Em7 | Em8 |
| *r_i_* | 8 | 6 | 1 | 4 | 2 | 7 | 3 | 5 |
| Int2🡪 Int2 | | | | | | | | |
| Emotion | Em1 | Em2 | Em3 | Em4 | Em5 | Em6 | Em7 | Em8 |
| *r_i_* | 4 | 2 | 5 | 8 | 1 | 3 | 7 | 6 |
| Int2🡪 Int3 | | | | | | | | |
| Emotion | Em1 | Em2 | Em3 | Em4 | Em5 | Em6 | Em7 | Em8 |
| *r_i_* | 1 | 6 | 7 | 3 | 8 | 2 | 5 | 4 |
| Int3🡪 Int1 | | | | | | | | |
| Emotion | Em1 | Em2 | Em3 | Em4 | Em5 | Em6 | Em7 | Em8 |
| *r_i_* | 8 | 4 | 1 | 2 | 3 | 6 | 5 | 7 |
| Int3🡪 Int2 | | | | | | | | |
| Emotion | Em1 | Em2 | Em3 | Em4 | Em5 | Em6 | Em7 | Em8 |
| *r_i_* | 8 | 6 | 5 | 7 | 3 | 4 | 1 | 2 |
| Int3🡪 Int3 | | | | | | | | |
| Emotion | Em1 | Em2 | Em3 | Em4 | Em5 | Em6 | Em7 | Em8 |
| *r_i_* | 1 | 2 | 7 | 3 | 6 | 4 | 8 | 5 |

^1^ The order of the probability from large to small.

**Table S8.** q-test results for transition probability of driving intention.

| q-test result for transition probability of Int1🡪 Int1. | | | | | r=2,  q_0.05_=2.8  q_0.01_=3.7  r=3,  q_0.05_=3.36  q_0.01_=4.2  r=4,  q_0.05_=3.68  q_0.01_=4.5  r=5,  q_0.05_=3.92  q_0.01_=4.71  r=6,  q_0.05_=4.1  q_0.01_=4.87  r=7,  q_0.05_=4.24  q_0.01_=5.01  r=8,  q_0.05_=4.36  q_0.01_=5.12 |
| --- | --- | --- | --- | --- | --- |
| Paired comparison | Difference value | *r* ^1^ | q value | Sig. |  |
| Em1:Em2 | -0.11 | 5 | -19.93 | *** |  |
| Em1:Em3 | -0.16 | 7 | -28.98 | *** |  |
| Em1:Em4 | -0.17 | 8 | -30.79 | *** |  |
| Em1:Em5 | -0.15 | 6 | -27.17 | *** |  |
| Em1:Em6 | -0.04 | 2 | -7.25 | *** |  |
| Em1:Em7 | -0.102 | 2 | -18.48 | *** |  |
| Em1:Em8 | -0.073 | 3 | -13.22 | *** |  |
| Em2:Em3 | -0.05 | 3 | -9.06 | *** |  |
| Em2:Em4 | -0.06 | 4 | -10.87 | *** |  |
| Em2:Em5 | -0.04 | 2 | -7.25 | *** |  |
| Em2:Em6 | 0.07 | 4 | 12.68 | *** |  |
| Em2:Em7 | 0.008 | 2 | 1.45 | - |  |
| Em2:Em8 | 0.037 | 3 | 6.70 | *** |  |
| Em3:Em4 | -0.01 | 2 | -1.81 | - |  |
| Em3:Em5 | 0.01 | 2 | 1.81 | - |  |
| Em3:Em6 | 0.12 | 6 | 21.74 | *** |  |
| Em3:Em7 | 0.058 | 4 | 10.51 | *** |  |
| Em3:Em8 | 0.087 | 5 | 15.76 | *** |  |
| Em4:Em5 | 0.02 | 3 | 3.62 | ** |  |
| Em4:Em6 | 0.13 | 7 | 23.55 | *** |  |
| Em4:Em7 | 0.068 | 5 | 12.32 | *** |  |
| Em4:Em8 | 0.097 | 6 | 17.57 | *** |  |
| Em5:Em6 | 0.11 | 5 | 19.93 | *** |  |
| Em5:Em7 | 0.048 | 3 | 8.69 | *** |  |
| Em5:Em8 | 0.077 | 4 | 13.95 | *** |  |
| Em6:Em7 | -0.062 | 3 | -11.23 | *** |  |
| Em6:Em8 | -0.033 | 2 | -5.98 | *** |  |
| Em7:Em8 | 0.029 | 2 | 5.25 | *** |  |
| q-test result for transition probability of Int1🡪 Int2. | | | | |  |
| Paired comparison | Difference value | *r* | q value | Sig. |  |
| Em1:Em2 | 0.08 | 2 | 8.23 | *** |  |
| Em1:Em3 | -0.05 | 6 | -5.15 | *** |  |
| Em1:Em4 | 0.09 | 3 | 9.26 | *** |  |
| Em1:Em5 | -0.02 | 2 | -2.06 | - |  |
| Em1:Em6 | -0.03 | 4 | -3.09 | - |  |
| Em1:Em7 | -0.028 | 4 | -2.88 | - |  |
| Em1:Em8 | -0.037 | 5 | -3.81 | - |  |
| Em2:Em3 | -0.13 | 7 | -13.38 | *** |  |
| Em2:Em4 | 0.01 | 2 | 1.03 | - |  |
| Em2:Em5 | -0.1 | 3 | -10.29 | *** |  |
| Em2:Em6 | -0.11 | 5 | -11.32 | *** |  |
| Em2:Em7 | -0.108 | 4 | -11.12 | *** |  |
| Em2:Em8 | -0.117 | 6 | -12.04 | *** |  |
| Em3:Em4 | 0.14 | 8 | 14.41 | *** |  |
| Em3:Em5 | 0.03 | 5 | 3.09 | - |  |
| Em3:Em6 | 0.02 | 3 | 2.06 | - |  |
| Em3:Em7 | 0.022 | 4 | 2.26 | - |  |
| Em3:Em8 | 0.013 | 2 | 1.34 | - |  |
| Em4:Em5 | -0.11 | 4 | -11.32 | *** |  |
| Em4:Em6 | -0.12 | 6 | -12.35 | *** |  |
| Em4:Em7 | -0.118 | 5 | -12.15 | *** |  |
| Em4:Em8 | -0.127 | 7 | -13.07 | *** |  |
| Em5:Em6 | -0.01 | 3 | -1.03 | - |  |
| Em5:Em7 | -0.008 | 2 | -0.82 | - |  |
| Em5:Em8 | -0.017 | 4 | -1.75 | - |  |
| Em6:Em7 | 0.002 | 2 | 0.21 | - |  |
| Em6:Em8 | -0.007 | 2 | -0.72 | - |  |
| Em7:Em8 | -0.009 | 3 | -0.93 | - |  |
| q-test result for transition probability of Int1🡪 Int3. | | | | |  |
| Paired comparison | Difference value | *r* | q value | Sig. |  |
| Em1:Em2 | 0.03 | 2 | 2.71 | - |  |
| Em1:Em3 | 0.21 | 8 | 18.94 | *** |  |
| Em1:Em4 | 0.08 | 4 | 7.21 | *** |  |
| Em1:Em5 | 0.17 | 7 | 15.33 | *** |  |
| Em1:Em6 | 0.07 | 3 | 6.31 | *** |  |
| Em1:Em7 | 0.13 | 3 | 11.72 | *** |  |
| Em1:Em8 | 0.11 | 5 | 9.92 | *** |  |
| Em2:Em3 | 0.18 | 7 | 16.23 | *** |  |
| Em2:Em4 | 0.05 | 3 | 4.51 | *** |  |
| Em2:Em5 | 0.14 | 6 | 12.62 | *** |  |
| Em2:Em6 | 0.04 | 2 | 3.61 | ** |  |
| Em2:Em7 | 0.1 | 5 | 9.02 | *** |  |
| Em2:Em8 | 0.08 | 4 | 7.21 | *** |  |
| Em3:Em4 | -0.13 | 5 | -11.72 | *** |  |
| Em3:Em5 | -0.04 | 2 | -3.61 | ** |  |
| Em3:Em6 | -0.14 | 6 | -12.62 | *** |  |
| Em3:Em7 | -0.08 | 3 | -7.21 | *** |  |
| Em3:Em8 | -0.1 | 4 | -9.02 | *** |  |
| Em4:Em5 | 0.09 | 4 | 8.12 | *** |  |
| Em4:Em6 | -0.01 | 2 | -0.90 | - |  |
| Em4:Em7 | 0.05 | 3 | 4.51 | *** |  |
| Em4:Em8 | 0.03 | 2 | 2.71 | - |  |
| Em5:Em6 | -0.1 | 5 | -9.02 | *** |  |
| Em5:Em7 | -0.04 | 2 | -3.61 | ** |  |
| Em5:Em8 | -0.06 | 3 | -5.41 | *** |  |
| Em6:Em7 | 0.06 | 4 | 5.41 | *** |  |
| Em6:Em8 | 0.04 | 3 | 3.61 | ** |  |
| Em7:Em8 | -0.02 | 2 | -1.80 | - |  |
| q-test result for transition probability of Int2🡪 Int1. | | | | |  |
| Paired comparison | Difference value | *r* | q value | Sig. |  |
| Em1:Em2 | -0.07 | 3 | -10.66 | *** |  |
| Em1:Em3 | -0.14 | 8 | -21.33 | *** |  |
| Em1:Em4 | -0.09 | 5 | -13.71 | *** |  |
| Em1:Em5 | -0.12 | 7 | -18.28 | *** |  |
| Em1:Em6 | -0.02 | 2 | -3.05 | ** |  |
| Em1:Em7 | -0.11 | 2 | -16.76 | *** |  |
| Em1:Em8 | -0.08 | 4 | -12.19 | *** |  |
| Em2:Em3 | -0.07 | 6 | -10.66 | *** |  |
| Em2:Em4 | -0.02 | 3 | -3.05 | - |  |
| Em2:Em5 | -0.05 | 5 | -7.62 | *** |  |
| Em2:Em6 | 0.05 | 2 | 7.62 | *** |  |
| Em2:Em7 | -0.04 | 4 | -6.09 | *** |  |
| Em2:Em8 | -0.01 | 2 | -1.52 | - |  |
| Em3:Em4 | 0.05 | 4 | 7.62 | *** |  |
| Em3:Em5 | 0.02 | 2 | 3.05 | ** |  |
| Em3:Em6 | 0.12 | 7 | 18.28 | *** |  |
| Em3:Em7 | 0.03 | 3 | 4.57 | *** |  |
| Em3:Em8 | 0.06 | 5 | 9.14 | *** |  |
| Em4:Em5 | -0.03 | 3 | -4.57 | *** |  |
| Em4:Em6 | 0.07 | 4 | 10.66 | *** |  |
| Em4:Em7 | -0.02 | 2 | -3.05 | ** |  |
| Em4:Em8 | 0.01 | 2 | 1.52 | - |  |
| Em5:Em6 | 0.1 | 6 | 15.24 | *** |  |
| Em5:Em7 | 0.01 | 2 | 1.52 | - |  |
| Em5:Em8 | 0.04 | 4 | 6.09 | *** |  |
| Em6:Em7 | -0.09 | 5 | -13.71 | *** |  |
| Em6:Em8 | -0.06 | 3 | -9.14 | *** |  |
| Em7:Em8 | 0.03 | 3 | 4.57 | *** |  |
| q-test result for transition probability of Int2🡪 Int2. | | | | |  |
| Paired comparison | Difference value | *r* | q value | Sig. |  |
| Em1:Em2 | -0.03 | 3 | -3.24 | - |  |
| Em1:Em3 | 0 | 2 | 0.00 | - |  |
| Em1:Em4 | 0.04 | 5 | 4.31 | ** |  |
| Em1:Em5 | -0.05 | 4 | -5.39 | *** |  |
| Em1:Em6 | -0.02 | 2 | -2.16 | - |  |
| Em1:Em7 | 0.03 | 2 | 3.24 | ** |  |
| Em1:Em8 | 0.01 | 3 | 1.08 | - |  |
| Em2:Em3 | 0.03 | 4 | 3.24 | - |  |
| Em2:Em4 | 0.07 | 7 | 7.55 | *** |  |
| Em2:Em5 | -0.02 | 2 | -2.16 | - |  |
| Em2:Em6 | 0.01 | 2 | 1.08 | - |  |
| Em2:Em7 | 0.06 | 6 | 6.47 | *** |  |
| Em2:Em8 | 0.04 | 5 | 4.31 | ** |  |
| Em3:Em4 | 0.04 | 4 | 4.31 | ** |  |
| Em3:Em5 | -0.05 | 5 | -5.39 | *** |  |
| Em3:Em6 | -0.02 | 3 | -2.16 | - |  |
| Em3:Em7 | 0.03 | 3 | 3.24 | - |  |
| Em3:Em8 | 0.01 | 2 | 1.08 | - |  |
| Em4:Em5 | -0.09 | 8 | -9.71 | *** |  |
| Em4:Em6 | -0.06 | 6 | -6.47 | *** |  |
| Em4:Em7 | -0.01 | 2 | -1.08 | - |  |
| Em4:Em8 | -0.03 | 3 | -3.24 | - |  |
| Em5:Em6 | 0.03 | 3 | 3.24 | - |  |
| Em5:Em7 | 0.08 | 7 | 8.63 | *** |  |
| Em5:Em8 | 0.06 | 6 | 6.47 | *** |  |
| Em6:Em7 | 0.05 | 5 | 5.39 | *** |  |
| Em6:Em8 | 0.03 | 4 | 3.24 | - |  |
| Em7:Em8 | -0.02 | 2 | -2.16 | - |  |
| q-test result for transition probability of Int2🡪 Int3. | | | | |  |
| Paired comparison | Difference value | *r* | q value | Sig. |  |
| Em1:Em2 | 0.1 | 6 | 8.87 | *** |  |
| Em1:Em3 | 0.14 | 7 | 12.42 | *** |  |
| Em1:Em4 | 0.05 | 3 | 4.44 | *** |  |
| Em1:Em5 | 0.17 | 8 | 15.08 | *** |  |
| Em1:Em6 | 0.04 | 2 | 3.55 | ** |  |
| Em1:Em7 | 0.08 | 2 | 7.10 | *** |  |
| Em1:Em8 | 0.07 | 4 | 6.21 | *** |  |
| Em2:Em3 | 0.04 | 2 | 3.55 | ** |  |
| Em2:Em4 | -0.05 | 4 | -4.44 | ** |  |
| Em2:Em5 | 0.07 | 3 | 6.21 | *** |  |
| Em2:Em6 | -0.06 | 5 | -5.32 | *** |  |
| Em2:Em7 | -0.02 | 2 | -1.77 | - |  |
| Em2:Em8 | -0.03 | 3 | -2.66 | - |  |
| Em3:Em4 | -0.09 | 5 | -7.99 | *** |  |
| Em3:Em5 | 0.03 | 2 | 2.66 | - |  |
| Em3:Em6 | -0.1 | 6 | -8.87 | *** |  |
| Em3:Em7 | -0.06 | 3 | -5.32 | *** |  |
| Em3:Em8 | -0.07 | 4 | -6.21 | *** |  |
| Em4:Em5 | 0.12 | 6 | 10.65 | *** |  |
| Em4:Em6 | -0.01 | 2 | -0.89 | - |  |
| Em4:Em7 | 0.03 | 3 | 2.66 | - |  |
| Em4:Em8 | 0.02 | 2 | 1.77 | - |  |
| Em5:Em6 | -0.13 | 7 | -11.54 | *** |  |
| Em5:Em7 | -0.09 | 4 | -7.99 | *** |  |
| Em5:Em8 | -0.1 | 5 | -8.87 | *** |  |
| Em6:Em7 | 0.04 | 4 | 3.55 | - |  |
| Em6:Em8 | 0.03 | 3 | 2.66 | - |  |
| Em7:Em8 | -0.01 | 2 | -0.89 | - |  |
| q-test result for transition probability of Int3🡪 Int1. | | | | |  |
| Paired comparison | Difference value | *r* | q value | Sig. |  |
| Em1:Em2 | -0.07 | 5 | -14.07 | *** |  |
| Em1:Em3 | -0.13 | 8 | -26.13 | *** |  |
| Em1:Em4 | -0.1 | 7 | -20.10 | *** |  |
| Em1:Em5 | -0.09 | 6 | -18.09 | *** |  |
| Em1:Em6 | -0.04 | 3 | -8.04 | *** |  |
| Em1:Em7 | -0.06 | 3 | -12.06 | *** |  |
| Em1:Em8 | -0.02 | 2 | -4.02 | *** |  |
| Em2:Em3 | -0.06 | 4 | -12.06 | *** |  |
| Em2:Em4 | -0.03 | 3 | -6.03 | *** |  |
| Em2:Em5 | -0.02 | 2 | -4.02 | *** |  |
| Em2:Em6 | 0.03 | 3 | 6.03 | *** |  |
| Em2:Em7 | 0.01 | 2 | 2.01 | - |  |
| Em2:Em8 | 0.05 | 4 | 10.05 | *** |  |
| Em3:Em4 | 0.03 | 2 | 6.03 | *** |  |
| Em3:Em5 | 0.04 | 3 | 8.04 | *** |  |
| Em3:Em6 | 0.09 | 6 | 18.09 | *** |  |
| Em3:Em7 | 0.07 | 5 | 14.07 | *** |  |
| Em3:Em8 | 0.11 | 7 | 22.11 | *** |  |
| Em4:Em5 | 0.01 | 2 | 2.01 | - |  |
| Em4:Em6 | 0.06 | 5 | 12.06 | *** |  |
| Em4:Em7 | 0.04 | 4 | 8.04 | *** |  |
| Em4:Em8 | 0.08 | 6 | 16.08 | *** |  |
| Em5:Em6 | 0.05 | 4 | 10.05 | *** |  |
| Em5:Em7 | 0.03 | 3 | 6.03 | *** |  |
| Em5:Em8 | 0.07 | 5 | 14.07 | *** |  |
| Em6:Em7 | -0.02 | 2 | -4.02 | *** |  |
| Em6:Em8 | 0.02 | 2 | 4.02 | *** |  |
| Em7:Em8 | 0.04 | 3 | 8.04 | *** |  |
| q-test result for transition probability of Int3🡪 Int2. | | | | |  |
| Paired comparison | Difference value | *r* | q value | Sig. |  |
| Em1:Em2 | -0.08 | 3 | -7.72 | *** |  |
| Em1:Em3 | -0.17 | 4 | -16.40 | *** |  |
| Em1:Em4 | -0.08 | 2 | -7.72 | *** |  |
| Em1:Em5 | -0.19 | 6 | -18.33 | *** |  |
| Em1:Em6 | -0.18 | 5 | -17.36 | *** |  |
| Em1:Em7 | -0.25 | 5 | -24.12 | *** |  |
| Em1:Em8 | -0.23 | 7 | -22.19 | *** |  |
| Em2:Em3 | -0.09 | 2 | -8.68 | *** |  |
| Em2:Em4 | 0 | 2 | 0.00 | - |  |
| Em2:Em5 | -0.11 | 4 | -10.61 | *** |  |
| Em2:Em6 | -0.1 | 3 | -9.65 | *** |  |
| Em2:Em7 | -0.17 | 6 | -16.40 | *** |  |
| Em2:Em8 | -0.15 | 5 | -14.47 | *** |  |
| Em3:Em4 | 0.09 | 3 | 8.68 | *** |  |
| Em3:Em5 | -0.02 | 3 | -1.93 | - |  |
| Em3:Em6 | -0.01 | 2 | -0.96 | - |  |
| Em3:Em7 | -0.08 | 5 | -7.72 | *** |  |
| Em3:Em8 | -0.06 | 4 | -5.79 | *** |  |
| Em4:Em5 | -0.11 | 5 | -10.61 | ** |  |
| Em4:Em6 | -0.1 | 4 | -9.65 | *** |  |
| Em4:Em7 | -0.17 | 7 | -16.40 | *** |  |
| Em4:Em8 | -0.15 | 6 | -14.47 | *** |  |
| Em5:Em6 | 0.01 | 2 | 0.96 | - |  |
| Em5:Em7 | -0.06 | 3 | -5.79 | *** |  |
| Em5:Em8 | -0.04 | 2 | -3.86 | *** |  |
| Em6:Em7 | -0.07 | 4 | -6.75 | *** |  |
| Em6:Em8 | -0.05 | 3 | -4.82 | *** |  |
| Em7:Em8 | 0.02 | 2 | 1.93 | - |  |
| q-test result for transition probability of Int3🡪 Int3. | | | | |  |
| Paired comparison | Difference value | *r* | q value | Sig. |  |
| Em1:Em2 | 0.15 | 2 | 13.26 | *** |  |
| Em1:Em3 | 0.3 | 7 | 26.52 | *** |  |
| Em1:Em4 | 0.18 | 3 | 15.91 | *** |  |
| Em1:Em5 | 0.28 | 6 | 24.75 | *** |  |
| Em1:Em6 | 0.22 | 4 | 19.45 | *** |  |
| Em1:Em7 | 0.31 | 4 | 27.40 | *** |  |
| Em1:Em8 | 0.25 | 5 | 22.10 | *** |  |
| Em2:Em3 | 0.15 | 6 | 13.26 | *** |  |
| Em2:Em4 | 0.03 | 2 | 2.65 | - |  |
| Em2:Em5 | 0.13 | 5 | 11.49 | *** |  |
| Em2:Em6 | 0.07 | 3 | 6.19 | *** |  |
| Em2:Em7 | 0.16 | 7 | 14.14 | *** |  |
| Em2:Em8 | 0.1 | 4 | 8.84 | *** |  |
| Em3:Em4 | -0.12 | 5 | -10.61 | *** |  |
| Em3:Em5 | -0.02 | 2 | -1.77 | - |  |
| Em3:Em6 | -0.08 | 4 | -7.07 | *** |  |
| Em3:Em7 | 0.01 | 2 | 0.88 | - |  |
| Em3:Em8 | -0.05 | 3 | -4.42 | *** |  |
| Em4:Em5 | 0.1 | 4 | 8.84 | *** |  |
| Em4:Em6 | 0.04 | 2 | 3.54 | ** |  |
| Em4:Em7 | 0.13 | 6 | 11.49 | *** |  |
| Em4:Em8 | 0.07 | 3 | 6.19 | *** |  |
| Em5:Em6 | -0.06 | 3 | -5.30 | *** |  |
| Em5:Em7 | 0.03 | 3 | 2.65 | - |  |
| Em5:Em8 | -0.03 | 2 | -2.65 | - |  |
| Em6:Em7 | 0.09 | 5 | 7.96 | *** |  |
| Em6:Em8 | -0.05 | 3 | -4.82 | *** |  |
| Em7:Em8 | 0.02 | 2 | 1.93 | - |  |

^1^ r=|r_i_-r_j_|+1
